# Anandamide and 2-arachidonoylglycerol baseline plasma concentrations and their clinical correlate in gambling disorder

**DOI:** 10.1192/j.eurpsy.2023.2460

**Published:** 2023-11-08

**Authors:** Isabel Baenas, Neus Solé-Morata, Roser Granero, Fernando Fernández-Aranda, Mitona Pujadas, Bernat Mora-Maltas, Ignacio Lucas, Mónica Gómez-Peña, Laura Moragas, Amparo del Pino-Gutiérrez, Javier Tapia, Rafael de la Torre, Marc N. Potenza, Susana Jiménez-Murcia

**Affiliations:** 1 Department of Clinical Psychology, Bellvitge University Hospital, Barcelona, Spain; 2 CIBER Physiopathology of Obesity and Nutrition (CIBEROBN), Instituto de Salud Carlos III, Barcelona Spain; 3 Psychoneurobiology of Eating and Addictive Behaviors Group, Neuroscience Program, Bellvitge Biomedical Research Institute (IDIBELL), L’Hospitalet de Llobregat, Barcelona, Spain; 4 Doctoral Program in Medicine and Translational Research, University of Barcelona, Barcelona, Spain; 5 Department of Psychobiology and Methodology, Autonomous University of Barcelona, Barcelona, Spain; 6 Department of Clinical Sciences, School of Medicine and Health Sciences, University of Barcelona, Barcelona, Spain; 7 Integrative Pharmacology and Systems Neuroscience Research Group, Hospital del Mar Research Institute (IMIM), Barcelona, Spain; 8 Department of Public Health, Mental Health and Perinatal Nursing, School of Nursing, University of Barcelona, Barcelona, Spain; 9 Department of Experimental and Health Sciences, Universitat Pompeu Fabra (CEXS-UPF), Barcelona, Spain; 10 Department of Psychiatry, Yale University School of Medicine, New Haven, CT, USA; 11 Child Study Center, Yale University School of Medicine, New Haven, CT, USA; 12 Connecticut Mental Health Center, New Haven, CT, USA; 13 Connecticut Council on Problem Gambling, Wethersfield, CT, USA; 14 Department of Neuroscience, Yale University, New Haven, CT, USA

**Keywords:** 2-arachidonoylglycerol, addictive behaviors, anandamide, gambling disorder, impulsive behaviors

## Abstract

**Introduction:**

Different components of the endocannabinoid (eCB) system such as their most well-known endogenous ligands, anandamide (AEA) and 2-arachidonoylglycerol (2-AG), have been implicated in brain reward pathways. While shared neurobiological substrates have been described among addiction-related disorders, information regarding the role of this system in behavioral addictions such as gambling disorder (GD) is scarce.

**Aims:**

Fasting plasma concentrations of AEA and 2-AG were analyzed in individuals with GD at baseline, compared with healthy control subjects (HC). Through structural equation modeling, we evaluated associations between endocannabinoids and GD severity, exploring the potentially mediating role of clinical and neuropsychological variables.

**Methods:**

The sample included 166 adult outpatients with GD (95.8% male, mean age 39 years old) and 41 HC. Peripheral blood samples were collected after overnight fasting to assess AEA and 2-AG concentrations (ng/ml). Clinical (i.e., general psychopathology, emotion regulation, impulsivity, personality) and neuropsychological variables were evaluated through a semi-structured clinical interview and psychometric assessments.

**Results:**

Plasma AEA concentrations were higher in patients with GD compared with HC (*p* = .002), without differences in 2-AG. AEA and 2-AG concentrations were related to GD severity, with novelty-seeking mediating relationships.

**Conclusions:**

This study points to differences in fasting plasma concentrations of endocannabinoids between individuals with GD and HC. In the clinical group, the pathway defined by the association between the concentrations of endocannabinoids and novelty-seeking predicted GD severity. Although exploratory, these results could contribute to the identification of potential endophenotypic features that help optimize personalized approaches to prevent and treat GD.

## Introduction

Gambling disorder (GD) is a psychiatric disorder characterized by a recurrent betting behavior despite negative consequences [1]. It has been classified within the “substance-related and addiction disorders” category in the *Diagnostic and Statistical Manual of Mental Disorders, Fifth Edition* (DSM-5), which recognizes the disorder as a behavioral addiction [1]. The estimated lifetime prevalence of GD varied between 0.2 and 10.6% worldwide, being around 1% in Spain [2–4]. It is considered as a complex disorder with a multifactorial etiology, involving psychosocial and biological factors [4, 5]. In this line, some studies have pointed to the role of different neuroendocrine systems in the pathogenesis of GD, which modulate neurobiological responses associated with reward and gratification [6–9].

Over the past few decades, the study of the endocannabinoid (eCB) system has garnered closed attention in the field of psychiatry due to its involvement in several brain functions such as cognition, emotion, impulsivity, and motivation [10]. Indeed, the eCB system has been directly implicated in the regulation of reward homeostasis in both animal and human studies [11–13]. This system includes endogenous ligands (i.e., eCBs), G-protein coupled cannabinoid receptors (CBRs), and enzymes to metabolize eCBs. Anandamide (AEA) and 2-arachidonoylglycerol (2-AG) are arguably the two most studied eCBs [14]. They are produced post-synaptically on demand to act on presynaptic CBRs, mainly types 1 and 2 (CB1R, CB2R). AEA and 2-AG do not seem fully interchangeable [15, 16], and even when interacting, they display specific functionality and/or act on different pathways regarding similar functions [15, 17]. AEA binds with slightly higher affinity to CB1R than CB2R, whereas 2-AG exhibits greater general agonist efficacy than AEA [14].

Although both types of receptors are located in the brain and peripheral tissues [18], CB1R has been predominantly found in the brain [19]. This receptor is widely distributed in mesolimbic structures integrated in the reward system (e.g., nucleus accumbens and ventral tegmental area) and other brain regions (e.g., prefrontal cortex, hippocampus, amygdala, insula, and hypothalamus) implicated in cue-elicited craving, relapse-like behaviors, and conditioning processes [14, 20, 21]. Through their union to CB1R, eCBs act as retrograde inhibitors of neurotransmitter release (both inhibitory, gamma-aminobutyric acid (GABA), and excitatory, glutamate (GLU)), with a modulatory role of other neurotransmission systems also involved in reward processing and addiction (e.g., dopaminergic, serotonergic, opioid systems) [22, 23].

The eCB system seems to enhance motivation for different natural and learned rewards (e.g., palatable food, sex, money, or drugs) by stimulating dopaminergic signaling [24, 25]. Indeed, the action of eCBs on CB1R regulates short- and long-term synaptic plasticity in areas related to reward processing, being dysfunctional changes in neuroplasticity linked to addiction [25]. Then, it is not surprising that the dysregulation of the eCB system signaling has been linked to substance and nonsubstance addictive behaviors (e.g., food, drugs, sex) [13, 14, 25].

In this context, eCBs have been proposed to influence dopamine-related positive reinforcement that mediates craving and impulsive reward-seeking behaviors [26]. Likewise, the eCB system seems to participate in negative-reinforcement-driven seeking behaviors through their involvement in learning and memory processes [25]. A dysfunctional eCB system signaling between the limbic system and areas such as the prefrontal cortex and amygdala may result in emotional processing and sensory perceptions underlying addiction. Such processes influence the acquisition of habit learning and conditioned responses relevant to progressive impairments in control that characterize addiction [23, 27].

Moreover, the eCB system has been linked to impulsivity and executive functioning albeit in different ways [28–30]. For example, circulating AEA concentrations have been related to advantageous decision-making and better cognitive flexibility while 2-AG concentrations have been linked to impaired cognitive flexibility and inhibitory response tendencies in a nonclinical population [31]. Likewise, the association of the eCB system with personality traits, such as novelty-seeking, has also been noted in previous research [28]. Additionally, the involvement of this system in emotion regulation has suggested a role in the pathogenesis of mood disturbances [32, 33]. That said, it is worth mentioning that impulsivity, poor cognitive processing, a dysfunctional personality structure (e.g., high novelty-seeking), and emotion dysregulation represent core features related to the pathogenesis of GD [4, 34–36].

Altogether, dysfunctions on the eCB system seem to be involved in several processes related to addiction [13, 25]. However, there is still controversy whether eCB system dysfunctions may be associated with causes and/or consequences of exposure to potentially addictive rewards. In fact, exposure to rewards has been proposed to induce, in turn, changes in the eCB system functioning [27]. In this sense, additional studies are needed to better understand the link between the eCB system and the pathogenesis of addiction. Nonetheless, preclinical and clinical evidence has led to the eCB system being proposed as a potential therapeutic target in psychiatry. In this line, CBR blockade or eCBs synthesis inhibition (e.g., enzyme inhibitors) seem to reduce reward consumption and craving, as well as subsequent relapse [14, 24, 37, 38].

To the best of our knowledge, this is the first study to assess eCBs in GD. Then, this study aimed to measure baseline AEA and 2-AG plasma concentrations in fasting among patients with GD, compared with healthy controls (HC). Moreover, through a path analysis model, associations between eCBs concentrations and GD severity were analyzed, considering the mediating role of clinical and neuropsychological variables. Based on previous data, we expected to find differences in eCBs concentrations between groups. Besides, we hypothesized a distinctive association between AEA and 2-AG with GD severity.

## Methods

### Participants

The final sample consisted of *n* = 166 treatment-seeking adult outpatients with a diagnosis of GD according to DSM-5 criteria [1], mostly men (*n* = 159, 95.8%) with a mean age of 39.13 years old (*SD* = 13.73). They were voluntarily recruited between April 2018 and September 2021 at the Behavioral Addictions Unit of Bellvitge University Hospital (Barcelona, Spain). A total of 41 HC were recruited via advertisement from the same catchment area. Exclusion criteria in both groups were the presence of an organic mental disorder, an intellectual disability, a neurodegenerative disorder, or an active psychotic disorder. Although a valid blood sample was available in all cases, 11 patients and 1 HC were excluded from the initial sample (*n* = 177 patients with GD and *n* = 42 HC) due to incomplete clinical data.

### Assessments

#### Biological measures

Blood samples were obtained after an overnight fasting of at least 8 hours. Blood was centrifuged at 1700 *g* in a refrigerated centrifuge (4°C) for over 20 minutes. Plasma was immediately separated and stored at −80°C until the analysis was carried out. AEA and 2-AG were analyzed by liquid chromatography-mass spectrometry (LC/MS–MS) using a previously validated method [39].

#### Clinical and neuropsychological measures

Clinical and neuropsychological variables were collected using standardized instruments, which are properly described in the Supplementary Material. Briefly, clinical variables were measured using the Spanish adaptation of the following questionnaires: South Oaks Gambling Screen (SOGS) [40, 41]; Diagnostic Questionnaire for Pathological Gambling According to DSM criteria [42, 43], Symptom Checklist-90-Revised (SCL-90-R) [44, 45]; Temperament and Character Inventory-Revised (TCI-R) [46, 47]; Impulsive Behavior Scale (UPPS-P) [48, 49]; and Difficulties in Emotion Regulation Strategies (DERS) [50, 51]. This last psychometric assessment was not systematically recorded in the HC group. Neuropsychological data has been collected by the following instruments: Wisconsin Card Sorting Test (WCST) [52]; Stroop Color and Word Test (SCWT) [53]; Trail Making Test (TMT) [54]; and Digits task of the Wechsler Memory Scale-Third Edition (WMS-III) [55].

Additionally, body mass index (BMI) and disorder-related variables, such as the age of onset and duration of GD, were collected in a semi-structured face-to-face clinical interview as described elsewhere [56].

### Procedure

All participants were evaluated at the Behavioral Addictions Unit of Bellvitge University Hospital (Barcelona, Spain). The data collection was conducted by a trained multidisciplinary team (psychologists, psychiatrists, nurses) with more than 25 years of experience in the field of GD. A comprehensive semi-structured clinical interview was conducted in a first session assessing all aspects related to gambling behavior. The psychometric assessment of clinical variables and the extraction of blood samples took place in a second session. Blood samples were analyzed at the Integrative Pharmacology and Systems Neuroscience research group-Hospital del Mar Research Institute (IMIM) (Barcelona, Spain). The neuropsychological evaluation was performed in a third session. All the measures were assessed prior to the beginning of specialized treatment for GD in our Unit.

### Ethics

The present study was conducted according to the Declaration of Helsinki. The Clinical Research Ethics Committee of Bellvitge University Hospital approved this study (ref. PR329/19 and PR338/17). Signed informed consent was obtained from all participants.

### Statistical analysis

Statistical analysis was conducted with Stata17 for Windows [57]. The between-group comparison of AEA and 2-AG concentrations was done with the analysis of covariance (ANCOVA), adjusting for sex, age, and BMI, as well as for sex and age regarding clinical variables (UPPS-P, SCL-90R, TCI-R, DERS). The standardized Cohen’s *d* coefficient measured the effect size for the mean comparisons (the thresholds of 0.20, 0.50, and 0.80 were considered for low, mild/moderate, and high-large effect sizes) [58].

Path analysis exploring the underlying relationships between variables among the GD subsample was performed through Structural Equation Models (SEM). The rationale for model specification was based on the background provided by the cumulated empirical evidence, with the condition of guaranteeing the clinical association of the relationships. All parameters were free-estimated, and parameters with no significant tests were deleted with the aim to obtain a final parsimonious model with the highest possible statistical power. Due to the large number of neuropsychological variables in the study, a latent variable was defined for the main measures obtained in each cognition test (WCST nonperseverative errors, WCST conceptual, TMT-A, TMT-B, Stroop interference, digits direct, and digits inverse). The goodness-of-fit was evaluated with standard criteria [59]: root mean square error of approximation RMSEA < 0.08, Bentler’s Comparative Fit Index CFI > 0.90, Tucker–Lewis Index TLI > 0.90, and standardized root mean square residual SRMR < 0.10. The global predictive capacity of the model was estimated with the coefficient of determination (CD).

## Results

### Description of the sample

In the clinical group, most participants were men (95.8%). The mean age was 39.1 years old (SD = 13.7), the mean age of GD onset was 27.8 years (SD = 11.9), and mean GD duration was 5.3 years (SD = 6.3). Most HC were men (90.2%), with a mean age of 49.3 years old (SD = 15.2). Table 1 displays the participants characteristics.

**Table 1.** Descriptive features of the sample

	Control *(n* = 41*)*		GD *(n* = 166*)*		
	*n*	%	*n*	%	*p*
Sex					
Women	4	9.8	7	4.2	.157
Men	37	90.2	159	95.8	

	Mean	SD	Mean	SD	*p*
Age (years-old)	49.27	15.23	39.13	13.73	<.001*
Age of onset of GD	–	–	27.81	11.95	
Duration of GD	–	–	5.30	6.27	

Abbreviations: GD, gambling disorder; SD, standard deviation.

* Significant comparison.

The presence of GD was associated with higher BMI, impulsivity (UPPS-P), general psychopathology (SLC-90R), novelty-seeking, and harm-avoidance (TCI-R), but lower self-directedness (TCI-R). Table 2 contains results of ANCOVA comparing clinical features between groups, adjusting for age and sex.

**Table 2.** Comparison of anthropometric, clinical, and neuropsychological variables between groups: ANCOVA

	Control (*n* = 41)		GD (*n* = 166)			
Adjusted for sex and age	Mean	SD	Mean	SD	*p*	*|d|*
BMI (kg/m^2^)	24.58	2.60	26.93	5.19	.004*	0.57^†^
UPPS-P: Lack of premeditation	20.84	4.02	24.27	6.11	.001*	0.66^†^
UPPS-P: Positive Urgency	20.26	5.85	31.93	10.39	<.00*	1.38^†^
UPPS-P: Negative Urgency	22.79	5.55	32.11	6.60	<.001*	1.53^†^
SCL-90R: GSI score	0.40	0.34	1.08	0.65	<.001*	1.31^†^
TCI-R: Novelty-seeking	98.42	10.63	111.29	12.23	<.001*	1.12^†^
TCI-R: Harm avoidance	87.69	17.86	99.17	17.15	<.001*	0.66^†^
TCI-R: Self-directedness	148.96	19.03	131.09	20.24	<.001*	0.91^†^
DERS: Total	–	–	90.68	21.37	–	–
WCST nonperseverative errors	9.15	10.42	17.46	13.09	<.001*	0.70^†^
WCST conceptual	66.37	8.66	62.40	14.56	.108	0.33
TMT A	27.65	8.10	30.44	9.85	.096	0.31
TMT B	62.12	22.07	79.27	41.47	.013*	0.52^†^
STROOP interference	3.29	7.71	3.02	6.89	.830	0.04
Digits direct	9.39	1.93	9.03	1.95	.311	0.19
Digits inverse	6.94	1.89	6.16	1.65	.013*	0.44

*Note: –,* Not available for this group.

Abbreviations: GD, gambling disorder; SD, standard deviation.

* Significant comparison.

† Effect size into ranges mild–moderate (|*d*| > 0.50) to high-large (|*d*| > 0.80).

### Comparison of eCBs concentrations between groups

**Figure 1.**
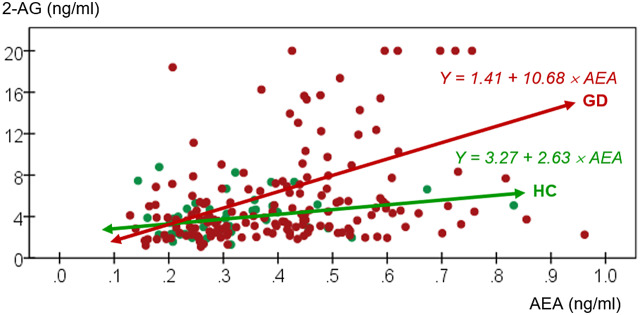
Scatterplot with individual data point concentrations of eCBs. 2-AG, 2-arachidonoylglycerol (ng/ml); AEA, anandamide (ng/ml); GD, gambling disorder; HC, healthy control.

Broadly, the clinical group displayed higher mean AEA concentrations while no between-group differences were found in 2-AG concentrations, after adjusting for age, sex, and BMI. Table 3 shows comparison of eCBs concentrations between groups. Additionally, Figure 1 displays a scatterplot with individual data point concentrations of eCBs.

**Table 3.** Comparison of endocannabinoids concentrations between groups: ANCOVA

	Control (*n* = 41)		GD (*n* = 166)			
Adjusted for sex, age, and body mass index	Mean	SD	Mean	SD	*p*	*|d|*
2-AG (ng/ml)	4.25	1.94	5.71	5.22	.100	0.37
AEA (ng/ml)	0.32	0.14	0.41	0.16	.002*	0.60^†^

Abbreviations: 2-AG, 2-arachidonoylglycerol (ng/ml); AEA, *N*-arachidonoylethanolamide (anandamide) (ng/ml); GD, gambling disorder; SD, standard deviation.

* Significant comparison.

† Effect size into ranges mild–moderate (|*d*| > 0.50) to high-large (|*d*| > 0.80).

### Path analysis

**Figure 2.**
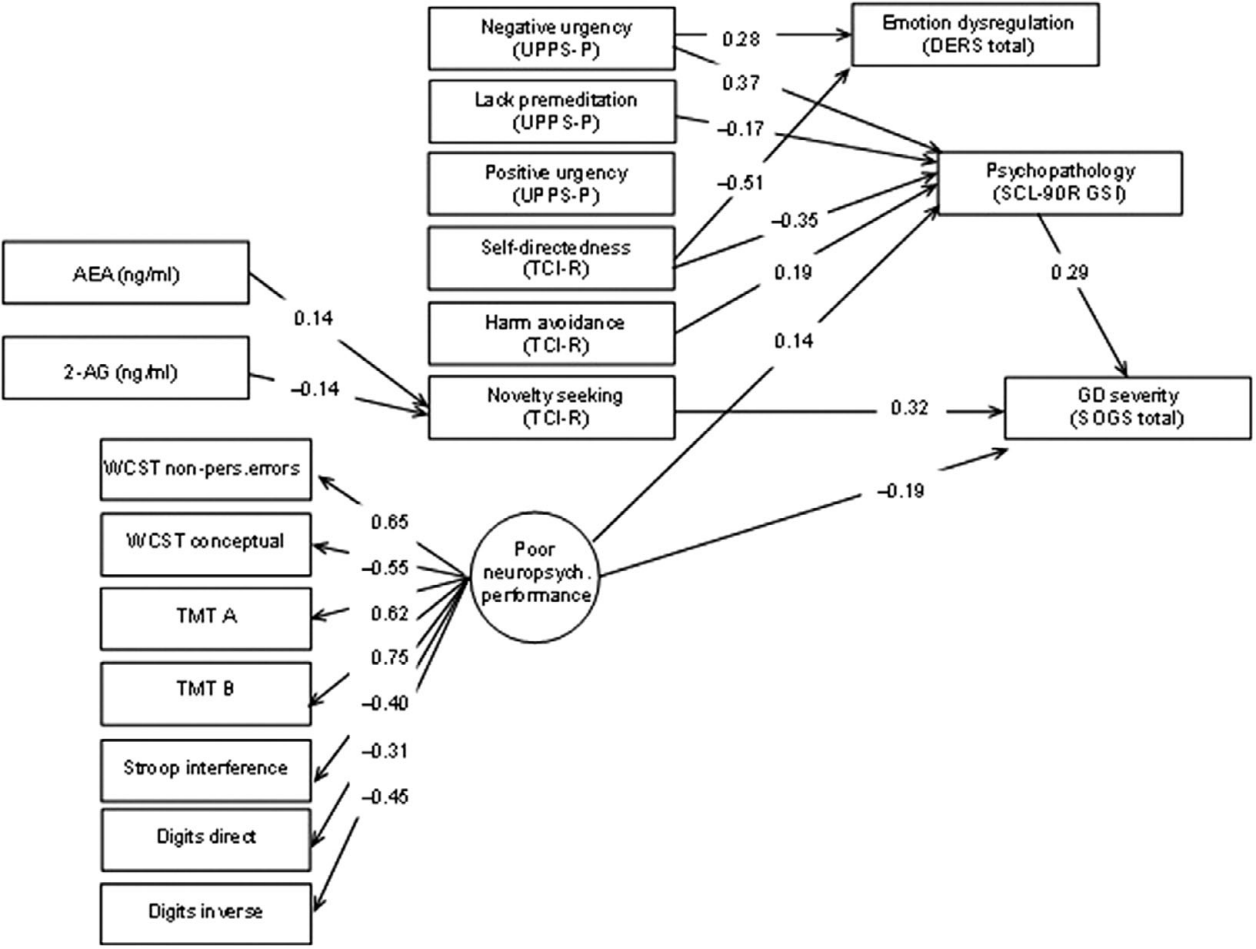
Path diagram with the standardized coefficients within the GD subsample. Results adjusted for sex and age. Only significant parameters are retained. Fit statistics: RMSEA = 0.065 (95%CI: 0.051 to 0.079); CFI = 0.909; TLI = 0.906; SRMR = 0.099; CD = 0.256.

Figure 2 displays the path diagram with the standardized coefficients of the SEM obtained in the GD group. Supplementary Table S1 contains the complete SEM results. This final model achieved adequate goodness-of-fit: RMSEA = 0.065 (95% confidence interval: 0.051 to 0.079), CFI = 0.909, TLI = 0.906 and SRMR = 0.099. The global predictive capacity was 26% (CD = 0.256).

All variables used to define the latent variable measuring the neuropsychological performance level achieved significant results. The signal of the measurement coefficients indicates that the higher the level in the latent variable, the higher the cognitive impairment.

Higher GD severity (SOGS) was directly associated with higher novelty-seeking (TCI-R) and psychopathological distress (SCL-90R, GSI), but lower neuropsychological dysfunction (latent variable). Indirect mediating links that contributed to increase the likelihood of higher GD severity were identified: (a) higher AEA and lower 2-AG concentrations were positively associated with higher novelty-seeking; (b) the path characterized by higher scores in harm avoidance and negative urgency as well as lower self-directedness and lack of premeditation was linked to higher scores in general psychopathology; and (c) worse cognitive performance was associated with more general psychopathology.

## Discussion

This exploratory study examined circulating AEA and 2-AG concentrations in individuals with and without GD. Moreover, relationships between those eCBs concentrations and GD severity were assessed through a SEM model that included different clinical and neuropsychological features. Broadly, the socio-demographic, clinical, and neuropsychological profile that characterized the clinical group was in line with previous works [60–62]. AEA concentrations were higher in individuals with GD than in HC after adjusting for sex, age, and BMI. The SEM analysis showed an association between eCBs concentrations and GD severity, with novelty-seeking being a mediating factor. Although it was not a primary objective of this study, the association between clinical and neuropsychological variables and GD severity was previously described [63–65].

Bearing in mind that this is a cross-sectional study, differences in eCBs concentrations between groups suggest an involvement of the eCB system in the pathogenesis of GD. Precisely, higher AEA concentrations in individuals with GD might indicate a dysfunctional eCB system, which has already been involved with the reinforcement of rewarding behaviors in other psychiatric conditions, such as substance use disorders or binge eating disorder [12, 16, 17, 27, 66–72]. In contrast to AEA, we did not observe statistically significant differences in 2-AG concentrations between groups. A possible hypothesis may be that 2-AG is highly sensitive to cue exposure, as previously proposed [69, 72, 73]. Moreover, it is worth mentioning that a prominent influence of preanalytical conditions on the plasma 2-AG concentrations (e.g., food intake, collection, and processing of blood samples) could be affecting our results [74]. Therefore, these aspects should be considered in the design of future studies.

Alternatively, the existence of compensatory mechanisms of the eCB system in efforts to counterbalance a dysfunctional activity could contribute to explain our results [26, 75]. This rationale may be reinforced by 2-AG acting as a full agonist on CB1R in contrast with the partial agonism of AEA, which gives AEA a higher modulatory capacity on CBRs and the eCB system’s activity, including other eCBs [14, 76]. Indeed, Maccarrone et al. [76] reported that elevated AEA concentrations could mediate a reduced biosynthesis of 2-AG in brain areas related to reward processing in an animal model, suggesting efforts to balance the eCB system tone. Furthermore, genetic studies have suggested that dysfunction of the enzymatic machinery could modulate eCBs concentrations. For example, the inhibition of the fatty acid amide hydrolase (FAAH), an enzyme involved in the degradation of AEA, has been related to increased AEA concentrations, but to an absence of significant changes in 2-AG or even, to decreased concentrations [27, 71, 76–78]. Altogether, these possibilities represent some of many factors that could underlie a dysfunctional eCB system and serve as a basis for future studies.

In the SEM analysis, we observed that higher AEA concentrations, but lower 2-AG, predicted higher GD severity through its association with novelty-seeking. While the relationship between novelty-seeking and GD severity has been previously established [65, 79–81], interestingly, a previous study reported a link between the eCB system and novelty-seeking [28]. Physiologically, the eCB system has been directly related to the balance between novelty-seeking and behavioral inhibition due to its regulation of GLU and GABA neurotransmission [82]. That is, a reduction in GLU transmission linked to the activation of the eCB system has been associated with decreased behavioral inhibition and increased novelty-seeking [82–84]. AEA has been described as a primary eCB involved in the control of the excitability of striatal neurons through a direct depressant action at GLU synapses, while it indirectly interferes with GABA inputs regulated by 2-AG [76, 85, 86]. That said, the relationship between the eCB system and novelty-seeking needs to be further explored, as well as its association with impulsivity-related measures and severity in GD.

Another important aspect to mention is that we assessed peripheral eCBs concentrations. Thus, it is possible that our results reflected an altered crosstalk between central and peripheral tissues, involving both top-down (initiated in the central nervous system) and bottom-up signaling (initiated in peripheral tissues) [87, 88]. Here, on the one hand, we wonder to what extent peripheral eCBs concentrations may be driven by CB1R-dependent signaling originated in the central nervous system and linked to changes in neuroplasticity. On the other hand, stimuli-mediated disturbances in peripheral eCBs concentrations could concurrently influence central hedonic processing.

While their lipidic nature facilitates crossing the blood–brain barrier [15], the involvement of peripheral eCBs in the pathophysiology of addiction-related disorders could also be mediated by the existence of a gut–brain–vagal axis [89]. In this respect, the origin of peripheral eCBs is an unresolved issue, although some authors speculate a predominantly gut origin [68]. Nonetheless, the involvement of peripheral tissues in the eCB system functioning at a central level seems more delineated for processes such as feeding regulation and energy homeostasis than for reward processing beyond food [89–91]. Taking into account that CB1R is found in peripheral tissues [18], future studies are needed to corroborate the implication of peripheral eCBs-CBR interactions in addictive-related behaviors other than food. Moreover, it would be interesting to examine whether other CBR such as CB2R may play a role in peripheral eCBs-CBR interactions due to its high concentrations in peripheral tissues including the hematological system [92].

Finally, some limitations should be highlighted. Individuals with GD were treatment-seeking patients from a hospital setting, which could limit the representativeness and generalizability of the results. Likewise, the cross-sectional nature of this study does not allow for drawing causal inferences. Moreover, the analysis of peripheral eCBs concentrations did not allow to infer the central functioning of the system. Therefore, future longitudinal studies exploring central correlates are warranted. Some strengths are also worth noting such as the inclusion of a control group, adjusting for potential confounding factors, and the use of a previously validated procedure to obtain eCBs concentrations.

## Conclusions

This is the first observational study focused on exploring the eCB system in a clinical sample with GD. We observed significant differences in fasting AEA plasma concentrations between individuals with GD and HC, being higher in the clinical group after adjusting for sex, age, and BMI. Moreover, the SEM analysis revealed interesting clinical correlates. Specifically, eCBs concentrations and novelty-seeking defined a pathway associated with a more severe profile among individuals with GD. Although these results might preliminarily contribute to shed light on the neurobiological mechanisms underlying GD severity, future research is warranted to further elucidate the role of the eCB system at a central level, as well as potential causality relationships.

## Supporting information

Baenas et al. supplementary material Baenas et al. supplementary material

Baenas et al. supplementary material Baenas et al. supplementary material

## Supplementary material

The supplementary material for this article can be found at https://doi.org/10.1192/j.eurpsy.2023.2460.
