# Supplementary material for: Anandamide and 2-arachidonoylglycerol baseline plasma concentrations and their clinical correlate in gambling disorder
Source: Eur Psychiatry. 2023 Nov 8;66(1):e97. doi: 10.1192/j.eurpsy.2023.2460 (PMC10755577; doi:10.1192/j.eurpsy.2023.2460)
Supplement: Baenas et al. supplementary material [file S0924933823024604sup001.docx]

**Supplementary Material**

**Appendix**

*Clinical measures*

The Spanish adaptation [1] of the *South Oaks Gambling Screen (SOGS)* [2] is a 20-item screening instrument for past-year gambling problems and related negative consequences. The total score obtained as the sum of the scored items has been used as a measure of problem-gambling severity, with a score of 5 or more suggestive of “probable pathological gambling.” The internal consistency in the study sample was good (Cronbach’s alpha *(α)*=.827).

The Spanish adaptation [3] of the *Diagnostic Questionnaire for Pathological Gambling According to DSM criteria* [4] is a self-report questionnaire with 19 items coded in a binary (yes-no) fashion, used for diagnosing GD according to the DSM-IV-TR [5] and DSM-5 criteria [6]. Our internal consistency was excellent (*α* = .917).

The Spanish adaptation [7] of the *Symptom Checklist-90-Revised (SCL-90-R)* [8] is a 90-item self-report questionnaire measured on an ordinal 3-point scale. It evaluates a broad range of psychological problems and psychopathology based on nine primary symptom dimensions (Somatization, Obsession-Compulsion, Interpersonal Sensitivity, Depression, Anxiety, Hostility, Phobic Anxiety, Paranoid Ideation, and Psychoticism). It includes three global indices (global severity index, positive symptom distress index, and total positive symptom). The internal consistency in our sample was excellent *(α* =.979).

The Spanish adaptation [9] of the *Temperament and Character Inventory-Revised* *(TCI-R)* [10] assesses with 240-items scored on a 5-point Likert scale personality tendencies related to three character dimensions (Self-Directedness, Cooperativeness, and Self-Transcendence) and four temperament dimensions (Harm Avoidance, Novelty Seeking, Reward Dependence and Persistence). The internal consistency in the study was between *α* =.704 (novelty-seeking) and *α* =.893 (persistence).

The Spanish adaptation [11] of the *Impulsive Behavior Scale* *(UPPS-P)* (Whiteside et al., 2005) measures five facets of impulsive behavior (negative urgency, positive urgency, lack of premeditation, lack of perseverance, and sensation-seeking) through 59 self-reported items. Our internal consistency was between *α* =.806 (lack of perseverance) and *α* =.942 (positive urgency).

The Spanish adaptation [13] of the *Difficulties in Emotion Regulation Strategies (DERS)* [14] is a 36-item self-reported scale assessing emotion dysregulation. It includes six subscales (i.e., non-acceptance of emotional responses, difficulties engaging in goal-directed behavior when having strong emotions, impulse-control difficulties, lack of emotional awareness, limited access to ER strategies, and lack of emotional clarity). Participants are asked to respond to each item using a five-point Likert scale ranging from 1 (almost never) to 5 (almost always). Higher scores reflect greater ER concerns. The internal consistency of the DERS total score in our sample was excellent (*α* = .934).

Consistency in the study (Cronbach-alpha):

| *Psychopathology: SCL-90R* |  | *Impulsivity: UPPS-P* |  | *Personality: TCI-R* |  |
| --- | --- | --- | --- | --- | --- |
| Somatization | 0.862 | Lack premeditation | 0.848 | Novelty seeking | 0.704 |
| Obsessive/compulsive | 0.863 | Lack perseverance | 0.806 | Harm avoidance | 0.868 |
| Interpersonal sensitivity | 0.842 | Sensation seeking | 0.882 | Reward dependence | 0.760 |
| Depressive | 0.921 | Positive urgency | 0.942 | Persistence | 0.893 |
| Anxiety | 0.873 | Negative urgency | 0.857 | Self-directedness | 0.885 |
| Hostility | 0.855 | Total | 0.934 | Cooperativeness | 0.832 |
| Phobic anxiety | 0.793 | *Emotional regulation: DERS* |  | Selt-transcendence | 0.849 |
| Paranoid Ideation | 0.774 | Non acceptance emotions | 0.886 |  |  |
| Psychotic | 0.865 | Diff. directed behaviors | 0.819 |  |  |
| Global indexes | 0.979 | Impulse control diff. | 0.828 |  |  |
| *Gambling severity* |  | Lack emotional awareness | 0.705 |  |  |
| DSM-5 criteria | 0.917 | Limited access emotions | 0.884 |  |  |
| SOGS questionnaire | 0.827 | Lack emotional clarity | 0.777 |  |  |
| *Food addiction: YFAS-2* | 0.974 | Total score | 0.934 |  |  |

*Neuropsychological measures*

The *Wisconsin Card Sorting Test (WCST)* [15] assesses cognitive flexibility. It includes four stimulus cards and 128 response cards, each showing different shapes, colors, and numbers of figures. Participants match response cards with stimulus cards in a way that it seems justifiable before receiving feedback (i.e., correct, or incorrect). After ten sequential correct answers the categorization criteria change. The number of complete categories, percentage of perseverative errors, and percentage of non-perseverative errors are recorded.

The *Stroop Color Word Test (SCWT)* [16] consists of three different lists beginning with a word list containing the names of colors printed in black ink followed by a color list that comprises letter *“X”* printed in color and, finally, by a color-word list constituted of names of colors in colored ink that matches or does not match the written name. Three final scores are obtained based on the number of items that the participant can read on each of the three lists in a time of 45 seg. It assesses tendencies to inhibit cognitive interference, which occurs when the processing of a stimulus feature affects the simultaneous processing of another attribute of the same stimulus.

The *Trail Making Test (TMT)* [17] consists of 25 circles spread out over two sheets of paper (Parts A and B). Participants are instructed to connect these circles drawing a line between consecutive numbers (part A) and alternating numbers and letters following a sequential order (part B). The task assesses visual conceptual and visual-motor tracking, entailing motor speed, attention, and set-shifting involving alternation between cognitive categories. Each part is scored according to the time spent to complete the task.

The *Digits task* of the *Wechsler Memory Scale-Third Edition (WMS-III)* [18] consists of two lists of digits presented verbally by the examiner. In the Digits Forward Task (first list), the participant is asked to repeat the digits in the same order. It assesses short-term memory and attention skills. In the Digits Backward Task (second list), the participant is asked to repeat the digits in reverse order. It evaluates verbal working memory due to internal manipulation of mnemonic representations of verbal information in the absence of external cues.

**References**

[1] Echeburúa E, Báez C, Fernández-Montalvo J, Páez D, Baez C, Fernández-Montalvo J, et al. Cuestionario de Juego Patológico de South Oaks (SOGS): validación española. Análisis y Modif La Conduct 1994;20:769–91.

[2] Lesieur HR, Blume SB. The South Oaks Gambling Screen (SOGS): A new instrument for the identification of Pathological gamblers. Am J Psychiatry 1987;144:1184–8. https://doi.org/10.1176/ajp.144.9.1184.

[3] Jiménez-Murcia S, Stinchfield R, Álvarez-Moya E, Jaurrieta N, Bueno B, Granero R, et al. Reliability, validity, and classification accuracy of a spanish translation of a measure of DSM-IV diagnostic criteria for pathological gambling. J Gambl Stud 2009;25:93–104. https://doi.org/10.1007/s10899-008-9104-x.

[4] Stinchfield R. Reliability, Validity, and Classification Accuracy of a Measure of DSM-IV Diagnostic Criteria for Pathological Gambling. Am J Psychiatry 2003;160:180–2. https://doi.org/10.1176/appi.ajp.160.1.180.

[5] American Psychiatric association (APA). Diagnostic and Statistical Manual of Mental Disorders, 4th edition. 4th ed. Washington, DC: American Psychiatric Association; 2000.

[6] APA. American Psychiatric Association. Diagnostic and Statistical Manual of Mental Disorders, Washington DC 2013.

[7] Derogatis LR. SCL-90-R. Cuestionario de 90 Síntomas-Manual. Madrid, Spain: 2002.

[8] Derogatis LR. SCL-90-R: Symptom Checklist-90-R. Administration, Scoring and Procedures Manuall—II for the Revised Version. Towson, MD, USA,: Clinical Psychometric Research:; 1994.

[9] Gutiérrez-Zotes JA, Bayón C, Montserrat C, Valero J, Labad A, Cloninger CR, et al. Temperament and Character Inventory Revised (TCI-R). Standardization and normative data in a general population sample. Actas Esp Psiquiatr 2004;32:8–15.

[10] Cloninger CR. The Temperament and Character Inventory—Revised. St Louis, MO, USA: Center for Psychobiology of Personality, Washington University; 1999.

[11] Verdejo-García A, Lozano Ó, Moya M, Alcázar MÁ, Pérez-García M. Psychometric properties of a spanish version of the UPPS-P impulsive behavior scale: Reliability, validity and association with trait and cognitive impulsivity. J Pers Assess 2010;92:70–7. https://doi.org/10.1080/00223890903382369.

[12] Whiteside, S. P., Lynam, D. R., Miller, J. D., & Reynolds SK, Whiteside SP, Lynam DR, Miller JD, Reynolds SK. Validation of the UPPS impulsive behaviour scale: A four-factor model of impulsivity. Eur J Pers 2005;19. https://doi.org/10.1002/per.556.

[13] Wolz I, Agüera Z, Granero R, Jiménez-Murcia S, Gratz KL, Menchón JM, et al. Emotion regulation in disordered eating: Psychometric properties of the difficulties in emotion regulation scale among spanish adults and its interrelations with personality and clinical severity. Front Psychol 2015;6:907. https://doi.org/10.3389/fpsyg.2015.00907.

[14] Gratz KL, Roemer L. Multidimensional Assessment of Emotion Regulation and Dysregulation: Development, Factor Structure, and Initial Validation of the Difficulties in Emotion Regulation Scale. J. Psychopathol. Behav. Assess., vol. 26, Kluwer Academic/Plenum Publishers; 2004, p. 41–54. https://doi.org/10.1023/B:JOBA.0000007455.08539.94.

[15] Grant DA, Berg E. A behavioral analysis of degree of reinforcement and ease of shifting to new responses in a Weigl-type card-sorting problem. J Exp Psychol 1948. https://doi.org/10.1037/h0059831.

[16] Golden CJ. Stroop Color and Word Test: A manual for clinical and experimental uses. Chicago: Stoelting 1978.

[17] Reitan RM. Validity of the Trail Making Test as an Indicator of Organic Brain Damage. Percept Mot Skills 1958. https://doi.org/10.2466/pms.1958.8.3.271.

[18] Wechsler D. Wechsler Memory Scale- (Third Ed.). Psychol Corp 1997.

**Supplementary Table**

***Table S1*** *Complete results obtained in the SEM*

| *Direct effects* |  |  | *Coeff* | *SE* | *z* | *p* | *StdCoeff* |
| --- | --- | --- | --- | --- | --- | --- | --- |
| Structural | DERS-total | Negative urgency | 0.8325 | 0.2310 | 3.60 | .001 | 0.2762 |
|  |  | TCI-R self-directedness | -0.4954 | 0.0753 | -6.58 | .001 | -0.5061 |
|  | SCL-90R-GSI | Negative urgency | 0.0337 | 0.0068 | 4.96 | .001 | 0.3664 |
|  |  | Lack premeditation | -0.0166 | 0.0062 | -2.69 | .007 | -0.1665 |
|  |  | TCI-R harm avoidance | 0.0066 | 0.0025 | 2.62 | .009 | 0.1876 |
|  |  | TCI-R self-directedness | -0.0105 | 0.0024 | -4.44 | .001 | -0.3522 |
|  |  | Poor neuropsyc.perform. | 1.0000 | *(constr)* |  |  | 0.1368 |
|  | SOGS-total | TCI-R novelty seek. | 0.0871 | 0.0190 | 4.57 | .001 | 0.3211 |
|  |  | SCL-90R-GSI | 1.5697 | 0.3654 | 4.30 | .001 | 0.2939 |
|  |  | Poor neuropsyc.perform. | -7.3827 | 4.7547 | -1.55 | .120 | -0.1891 |
|  | TCI-R novelty seeking | AEA | 10.4955 | 4.8296 | 2.17 | .030 | 0.1427 |
|  |  | 2-AG | -0.3322 | 0.1491 | -2.23 | .026 | -0.1449 |
| Measurement | Poor neuropsyc.perform. | WCST non-pers.errors | 102.5239 | 52.6982 | 1.95 | .052 | 0.6510 |
|  |  | WCST conceptual | -96.5024 | 50.9953 | -1.89 | .058 | -0.5524 |
|  |  | TMT-A | 73.8005 | 37.8136 | 1.95 | .051 | 0.6226 |
|  |  | TMT-B | 374.8514 | 193.0170 | 1.94 | .052 | 0.7510 |
|  |  | Stroop interference | -33.1836 | 17.9938 | -1.84 | .065 | -0.4001 |
|  |  | Digits inverse | -8.9156 | 4.8470 | -1.84 | .066 | -0.4489 |
|  |  | Digits direct | -7.2399 | 4.2831 | -1.69 | .091 | -0.3079 |
| *Indirect effects* |  |  | *Coeff* | *SE* | *Z* | *p* | *StdCoeff* |
| Structural | SOGS-total | Negative urgency | 0.0529 | 0.0163 | 3.25 | .001 | 0.1077 |
|  |  | Lack premeditation | -0.0261 | 0.0114 | -2.28 | .022 | -0.0489 |
|  |  | TCI-R harm avoidance | 0.0104 | 0.0046 | 2.25 | .025 | 0.0551 |
|  |  | TCI-R self-directedness | -0.0165 | 0.0053 | -3.09 | .002 | -0.1035 |
|  |  | Poor neuropsyc.perform. | 1.5697 | 0.3654 | 4.30 | .001 | 0.0402 |
|  |  | AEA | 0.9137 | 0.4656 | 1.96 | .050 | 0.0458 |
|  |  | 2-AG | -0.0289 | 0.0144 | -2.00 | .045 | -0.0465 |
| *Total effects* |  |  | *Coeff* | *SE* | *Z* | *p* | *StdCoeff* |
| Structural | DERS-total | Negative urgency | 0.8325 | 0.2310 | 3.60 | .001 | 0.2762 |
|  |  | TCI-R self-directedness | -0.4954 | 0.0753 | -6.58 | .001 | -0.5061 |
|  | SCL-90R-GSI | Negative urgency | 0.0337 | 0.0068 | 4.96 | .001 | 0.3664 |
|  |  | Lack premeditation | -0.0166 | 0.0062 | -2.69 | .007 | -0.1665 |
|  |  | TCI-R harm avoidance | 0.0066 | 0.0025 | 2.62 | .009 | 0.1876 |
|  |  | TCI-R self-directedness | -0.0105 | 0.0024 | -4.44 | .001 | -0.3522 |
|  |  | Poor neuropsyc.perform. | 1.0000 | *(constr)* |  |  | 0.1368 |
|  | SOGS-total | TCI-R novelty seeking | 0.0871 | 0.0190 | 4.57 | .001 | 0.3211 |
|  |  | Negative urgency | 0.0529 | 0.0163 | 3.25 | .001 | 0.1077 |
|  |  | SCL-90R-GSI | 1.5697 | 0.3654 | 4.30 | .001 | 0.2939 |
|  |  | Lack premeditation | -0.0261 | 0.0114 | -2.28 | .022 | -0.0489 |
|  |  | TCI-R harm avoidance | 0.0104 | 0.0046 | 2.25 | .025 | 0.0551 |
|  |  | TCI-R self-directedness | -0.0165 | 0.0053 | -3.09 | .002 | -0.1035 |
|  |  | Poor neuropsyc.perform. | -5.8130 | 4.7228 | -1.23 | .218 | -0.1489 |
|  |  | AEA | 0.9137 | 0.4656 | 1.96 | .050 | 0.0458 |
|  |  | 2-AG | -0.0289 | 0.0144 | -2.00 | .045 | -0.0465 |
|  | TCI-R novelty seeking | AEA | 10.4955 | 4.8296 | 2.17 | .030 | 0.1427 |
|  |  | 2-AG | -0.3322 | 0.1491 | -2.23 | .026 | -0.1449 |
| Measurement | Poor neuropsyc.perform. | WCST non-pers.errors | 102.5239 | 52.6982 | 1.96 | .050 | 0.6510 |
|  |  | WCST conceptual | -96.5024 | 50.9953 | -1.89 | .058 | -0.5524 |
|  |  | TMT-A | 73.8005 | 37.8136 | 1.96 | .050 | 0.6226 |
|  |  | TMT-B | 374.8514 | 193.0170 | 1.96 | .050 | 0.7510 |
|  |  | Stroop interference | -33.1836 | 17.9938 | -1.84 | .065 | -0.4001 |
|  |  | Digits inverse | -8.9156 | 4.8470 | -1.84 | .066 | -0.4489 |
|  |  | Digits direct | -7.2399 | 4.2831 | -1.69 | .091 | -0.3079 |

*Note.* Constr: constrained coefficient. Coeff: coefficient. SE: standard error. StadCoeff: standardized coefficient. Neuropsyc.perform: neuropsychological performance. Pers.errors: perseverative errors.
